# Method of Directing Second Dentition

**Published:** 1854-07

**Authors:** James Taylor

**Affiliations:** Cincinnati.


*Method of Directing Second Dentition.* By JAMES TAYLOR, M. D., D. D. S., Cincinnati.

"Since the progressive eruption of each series of teeth is one of the laws ordained by the Creator, why have so many authors on dentistry endeavored, by the spirit of their systems, to follow a different course? Why do we find so many *dentes excerti* among children of the wealthy class, whose dentition meets with the more attention, while they are of so rare occurrence among the indigent, that have very little recourse to medical aid, but rely on the goodness of nature. Is not a part of these vicious dentitions, which we so frequently meet with in society, owing to the unsound system adopted by dentists?"—*Delabarre.*

"An ounce of preventive is worth a pound of cure," is an old maxim. Irregular teeth appear also to have been of ancient date, for history informs us that Hercules, Thima c h and the daughters of Mithridate, all had two or three rows of teeth. From this we may rightly infer that supernumerary and the deciduous organs had a place also in their jaws. Among intelligent dental practitioners it is generally acceded that so long as nature does her duty right, she should be let alone. We fear, however, that many mistake the right for the wrong and offer a helping hand when the proffered aid is not only not needed, but absolutely hurtful. We see almost daily proof this.

The whole subject of directing and regulating second dentition is an important one, and the profession of late appear to have taken hold of the subject with a desire to elicit the best mode of practice.

In the directing of second dentition, in so far as the removal of the decidious organs are concerned, we know that it is difficnlt to lay down a certain rule of practice. There is much in practice which must be governed by circumstances. Yet we feel that as a general rule, it is better to let nature alone than to be constantly attempting to aid her. Delabarre on this subject very justly remarks that "it is only necessary that the surgeon dentist should, for the most part, be a tranquil observer of the shedding of teeth; for the temporaries frequently fall out of themselves, and where he thinks he ought to aid naturc, we see that he should only extract one at a time, whose place may be supplied by another; so that in following this course, he will not notch the dentune of a subject, when any of the adult teeth have failed, as they frequently do, to be developed."

The excellent views which Delabarre gives us in his treatise on second dentition are worthy of the most careful study of every dentist and physician.

His theory in relation to this operation of the econemy and that for the eruption of the deciduous organs, have perhaps more facts and correct physiological deductions to sustain them than any other.

The dental practitioner should not only be a "tranquil observer" of nature in the shedding of the deciduous and the eruption of the permanent teeth, but he should have made the development of the teeth a special study. And we think a careful study of the position of the pulp of the permanent organs with reference to the deciduous one about to be shed or removed, is of the utmost importance, and correct views also in relation to the accretion of the jaws, is essential to guide us aright in our diagnosis and practice.

The position generally given to the permanent organs with reference to the deciduous, before the eruption of the former and loss of the latter, is at the age of three or four years, as follows. We give Delabarre's description of their position in the inferior maxilla:

"Near the symphysis, perpendicularly and deeply underneath the temporary incisors, whose roots, at this period, are incomplete, we shall find the crowns of the central incisors of replacement. The lateral incisor is behind, situated obliquely from the outs:de of the inside of the jaw, and covers a third of the posterior labial part of the central incisor, and about a half of the cuspid or canine. This last is situated lower, more deeply and further on them than the one just mentioned, and lies obliquely on the medial line, so that the three teeth form a triangular group, resembling that which we make with our last three fingers, in touching an annular index bring thus the middle one above the rank. At a little distance from the cuspidi, and immediately between the roots of the temporary molars, we find the bicuspidi enveloped in their membranes, an appendage of which is carried backward to reach the gum, so that the bicuspidi appear to be covered by the crowns of the milk molars. Each kind of teeth thus ossified, can be distinguished by their points; the incisors may be known by the three small tubercles that surmount the cutting edge of each, the cuspides by one alone that forms the summit of a cone, the bicuspidi by two ossified points, and the molars by four. As ossification gradually covers the pulp or dental ganglion, their points are united and form a sort of chapeau that may easily be detached, though intimately united to it, but by vessels of so delicate a nature that the least effort breaks them.

We may understand the order in which each series of teeth is placed, by taking for a comparison the ellipsis described by the gums; then drawing in our imagination three horizontal lines upon it, which shall be parallel to it, and pass over the summits of the teeth in ossification; at four years after birth, we shall find the central incisors upon the level of the second molars, the lateral incisors upon that of the first molars and the bicuspidi; while the cuspidi occupy the lowest line; this is worthy of our special notice.

These two classes of teeth, the temporary and permanent, are therelore developed at nearly the same period; but it is only when the first adorns the mouth of the child, that nature directs all her efforts to those that are to succeed them.

This attentive anatomical observation has not only shown us that the crowns of the teeth are formed at a very early period, but also, how, from the time of their manifestation, they take their transverse dimensions, such as they should have through the whole course of life, and that the portion of the circle, which the six anteriors occupy in the jaw, is at first of very limited extent, in order to allow them to be arranged at the side of each other. Nature has so disposed of them that each covers about one-third of another, which is precisely such an arrangement as we observe in the tiles upon houses. This disposition gives them such an obliquity, that supposed lines traversing their greatest diameter would cut the maxillary bone obliquely.

This arrangement of the teeth is found in all jaws; it is symmetrical, it is constant, and may be observed in all young subjects; it is a law from which nature never can and never does depart; it is, in short, an ingenious means employed by her to supply the want of space. Many writers either have not appreciated the importance of these imbrications, or have not sufficiently studied, on different subjects, what they have pleased to consider as irregularities resulting from a mal-formrtion of the jaw. Many mistakes in practice have resulted from this error."

In considering the consequences of thc too early removal of the deciduous organs, we must bear in mind the arrrangement here given; the central incisors being on a horizontal line with the second molars. These are of course the permanent organs of which we are speaking. We have next a horizontal line a little lower down, and on this line we have the lareral incisors the first molars and bicuspidi, and still below this line we have the cuspidati,and these,as Delabarre remarks, lies obliquely on the medial line. With this view of the organs before their eruption and also before the loss of any of the deciduous set, we are prepared first to ask, what purpose does the deciduous organs subserve, independent of their use in the mastication of food, or does their presence have any effect in controlling the position and direction of the permanent teeth? We think there can be no doubt of this. First, because they are necessary to the continued expansion of the alveolar arch; and second, when lost they disturb that arrangement of the permanent germs which an All-Wise Providence has selected as the best.

In the second place we are prepared to ask, what is the effect of the too early removal of the teeth? Their early loss occasions a contraction of the arch into which the permanent teeth appear. Sometimes their too early removal may injure the permanent organ so that it will never appear, and it is primarily the cause of great irregularity of the teeth.

We will more particularly notice some of the bad effects of the removal of these teeth before nature directs their removal, and also explain what we think are the indications for their extraction.

The first and most common case which presents itself is the removal of the lateral incisors, to give space for the central incisors which appear to want more room than is made by the removal of the central.

For a time this may appear to have the desired effect, yet the mischief is buried beneath the gum, for the permanent central incisors occupy a contracted space in the alveolar arch, fill up almost entirely the space made by the removal of the four, and hence no room is allowed for the lateral incisors, and as these teeth, and indeed all the incisors above and below, most generally point inward, they take their position within the circle and are held in this position by the central incisors and the deciduous cuspidati which are yet firm.

The absorption of the roots of the deciduous teeth are generally accomplished just in accordance with the wants of the permanent organs, and forms the most natural opening for these teeth. It will be remarked, however, that these teeth pointing generally a littte inward, that, that portion of the roots of the deciduous teeth are most observable. Say now that the lateral incisor having been removed to give room for the permanent central, this and the permanent cuspid which lies on a lower line approximates, and the lateral incisor is forced more within the circle, and having no natural opening made by the absorption of the root of the deciduous tooth, it forms its socket in accordance with the direction which the tooth has been forced to take, and becomes, unless relieved by art, permanently irregular.

Parents often, very often bring in their children just as the central incisors are making their appearance, and because these appear to be taking a wrong position within the circle, and the space not sufficient for them where the deciduous had stood, they insist on having out the lateral incisors. These teeth, however, as they are more and more protruded, take their position more and more outwardly, until they are perhaps forming a larger circle than that occupied by the deciduous teeth.

It may be asked, are there no conditions rendering the extraction of the deciduous lateral incisors necessary to give room for the central permanent? We believe not; at least we should wait about the period for their natural shedding before we resort to such treatment. We would first see what nature would effect for herself, and give her as much time to labor in as possible.

Any apparent benefit which we may obtain by too early an interference, is almost always more than counterbalanced by increased difficulties in the eruption and position of the lateral incisors and cuspidati.

In forming our diagnosis as it regards the extraction of these teeth, we can not be so much governed by the age of the child as the period when the first teeth were shed and other conditions which present themselves. Say a child is presented that has just fairly erupted the central incisors, and it is eight or even nine years of age. Well, because our books say that the laterals are shed from seven to nine, it would be no excuse for their removal. We should recollect that the first being tardy the others may all be like backward in making their appearance, and this will generally be the case; so that the question should not be so much, how old is the child, but how long since such and such teeth were erupted? We imagine that much malpractice results from this oversight.

We have had a case within the last few days of the following kind, and we give it not because it is singular, but because it points out a course of practice which we think is specially indicated:

The case was a Miss, between seven and eight, whose central incisors inferior have made their appearance, and are fully developed, but the laterals were removed to give them room. On one side the deciduous cuspid and incisor stand within a line of each other; on the other side the space formed by the removal of the lateral, is but little diminished, and for this reason the deciduous second molar had been removed. The space therefore obtained by the removal of the molar has operated about as much on the deciduous cuspid as that made by the removal of the lateral incisor.

It is obvious from this case, and hundreds of like character, that as much good can be derived from the removal of one of the molars as the lateral incisor; and as the after difficulty is nothing comparatively, we think this is the preferable practice where either is absolutely demanded.

The bicuspidati occupy less space than the deciduous molars; they may not by this practice be thrown into a crowded condition, and if they are, the front and exposed part of the dunture is beautiful and perfect.

Should this practice occasion a crowded condition of the bicuspidati one could be removed, and this in nine cases out of ten is of essential benefit.

We shall continue this subject and give cases illustrating practice.

